# Identification of immune cells in benign and malignant thymus tumors: a Mendelian randomization study

**DOI:** 10.1097/MS9.0000000000004395

**Published:** 2025-11-25

**Authors:** Nuo Yan, Silin Wang, Liancheng Ruan, Yang Zhang, Deyuan Zhang, Sheng Hu, Wenxun Xiong, Wenxiong Zhang, Chuan Yao, Yiping Wei

**Affiliations:** a Department of Thoracic Surgery, Affiliated Hospital of Jiujiang University, Jiujiang, China; b Department of Thoracic Surgery, The Second Affiliated Hospital of Nanchang University, Nanchang, Jiangxi, China

**Keywords:** genome wide association study (GWAS), immune cells, immune microenvironment, Mendelian randomization, thymus tumors

## Abstract

**Objective:**

We aimed to identify potential causal immune cells and their phenotypes in benign and malignant thymic tumors from the FinnGen database using Mendelian randomization (MR).

**Methods:**

A two-sample MR approach was employed to evaluate the causal associations between 731 immune cell traits and the risk of benign and malignant thymomas using publicly available genetic datasets. Immune traits analyzed included median fluorescence intensity (MFI), relative cell count (RC), absolute cell count (AC), and morphological parameters (MP). Robustness of findings was assessed through sensitivity analyses, ensuring minimal heterogeneity and horizontal pleiotropy.

**Results:**

Four immune cell phenotypes, including CCR2 on CD14+ CD16+ monocytes (95% CI: 1.163–1.775, *P* = 0.0007) and transitional AC (95% CI: 1.176–3.232, *P* = 0.0095), were causally associated with benign thymomas and identified as risk factors. Conversely, naive-mature B cell AC (95% CI: 0.438–0.887, *P* = 0.0087) and natural killer (NK) AC (95% CI: 0.237–0.812, *P* = 0.0087) displayed protective effects. For malignant thymomas, seven immune phenotypes, including Memory B cell %B cell (95% CI: 1.160–2.242, *P* = 0.0045), were linked as risk factors, while three, such as IgD+ CD24− %B cells (95% CI: 0.438–0.868, *P* = 0.0055), exhibited protective associations. Sensitivity analyses confirmed the robustness of the results.

**Conclusion:**

Using genetic methodologies, we identified a robust link between immune cells and both benign and malignant thymic tumors. This study highlights the distinct immune phenotypes between benign and malignant thymic tumors, shedding light on their immunological mechanisms and suggesting new avenues for clinical immunotherapy.

## Introduction

Thymomas, mediastinal tumors derived from thymic epithelial cells, are among the most common primary tumors of the adult mediastinum^[1]^. Thymomas are classified by the World Health Organization (WHO) into types A, AB, B1, B2, and B3, with most exhibiting uncertain biological behavior and encoded as malignant (behavior code 3) according to the International Classification of Diseases for Oncology (ICD-O)^[2]^. Exceptions include micronodular thymoma with lymphoid stroma (code 1, borderline/uncertain) and lipofibroadenoma (code 0, benign). A priori malignant subtypes encompass thymic carcinomas, such as squamous cell carcinoma, lymphoepithelioma-like carcinoma, sarcomatoid carcinoma, clear cell carcinoma, basaloid carcinoma, and others^[3]^. Histologic heterogeneity is common, with many thymomas comprising multiple subtypes, which complicates diagnosis and patient selection in genetic studies. Thymomas are categorized as benign or malignant based on their pathological features and behavior^[2,4]^. Despite their low incidence (approximately 1.5 cases per million)^[5]^, thymic tumors have garnered immense interest in thoracic oncology and immunology owing to their unique anatomy, biological traits, and links to immune-related diseases, such as myasthenia gravis and autoimmune thyroid disorders^[6–8]^. Thymoma development is assumed to involve various genetic and immune factors; however, the exact mechanisms remain largely unexplored. Studies have suggested that patients with thymoma may display chromosomal instability and polygenic mutations, with a notably high mutation rate of the GTF2I gene in type A thymomas^[9]^. Environmental factors, such as radiation exposure may also contribute to the development of thymomas. The thymus is a vital immune organ that facilitates T-cell differentiation and maturation. It also plays crucial roles in maintaining immune tolerance and regulating homeostasis. Tumor development can disrupt immune function through diverse mechanisms, creating a complex tumor microenvironment (TME)^[10]^. The TME not only affects tumor initiation and progression, but also significantly affects patient responses to therapy. Recently, immune checkpoint inhibitors (e.g., anti-PD-1/PD-L1 antibodies) have been increasingly applied in treating thymoma and thymic carcinoma^[11,12]^. Thus, investigating the immunological features of thymic tumors, particularly their immune cell composition and function within the TME, is important.

The role of immune cells in the TME has become a central focus in oncology research. Immune cells play crucial roles in tumor initiation, progression, and treatment responses by regulating immune surveillance, immune evasion, and inflammation^[13]^. Studies have shown that immune cells, including effector T, regulatory T (Tregs), and natural killer (NK) cells and macrophages, play major roles in immune evasion in various tumors^[14–16]^. However, the distribution and functional characteristics of immune cells in benign and malignant thymic tumors remain scarcely studied. Benign thymomas and malignant thymic carcinomas differ significantly in their pathological features, invasiveness, and biological behavior, with distinct immunological mechanisms that may affect the diagnosis and treatment strategies. Most current clinical research relies on observational methods, which are limited by confounding factors and reverse causality, hindering the accurate identification of the causal relationship between immune cells and thymic tumors. Furthermore, previous studies have focused primarily on individual immune cell types or specific signaling pathways without comprehensively exploring the synergistic effects of different immune cells in the TME or their relationship with tumor biology, leaving key gaps in causal inference for immune phenotypes. Mendelian randomization (MR) analysis offers a novel approach to overcome the limitations of observational studies^[17]^. MR leverages the natural grouping of genetic variations using genes as instrumental variables for causal inference, effectively reducing confounding factors and avoiding reverse causality^[18]^. This method has been widely applied in tumor immunology research, offering a reliable tool for exploring the causal relationships between immune cells and tumors^[19]^. Additionally, MR analysis based on large-scale genome-wide association studies (GWAS)^[20]^ can comprehensively analyze the role of different immune cell types in thymic tumors, providing strong evidence for revealing their immunological mechanisms. Thymoma’s immune environment is unique, featuring thymic epithelial disruption leading to autoimmunity (e.g., myasthenia gravis) and heightened NK and monocyte involvement, contrasting with PD-L1-dominant evasion in lung cancer or B cell–centric dysregulation in lymphoma^[21–23]^.

This study aimed to integrate GWAS and genetic data related to thymic tumors to construct genetic instrumental variables for immune cell types and to systematically explore causal inferences for key immune cell types in both benign and malignant thymic tumors using MR. Furthermore, by analyzing the differences in specific immune cells between benign and malignant thymic tumors, we explored the roles of immune cells and their immunological characteristics in both types of thymomas. This research will not only reveal new mechanisms underlying thymic tumor development but also provide a theoretical foundation for immunotherapy and precision medicine. This study was conducted without the use of artificial intelligence, in full compliance with the TITAN guidelines for transparency in AI reporting^[24]^.

**HIGHLIGHTS**

- Causal immune links via Mendelian Randomization: Two-sample MR analysis of 731 immune traits (MFI, RC, AC, MP) from GWAS data revealed robust causal associations with benign and malignant thymic tumors, confirmed by sensitivity analyses showing no heterogeneity or pleiotropy.
- Benign thymoma risk and protection: Four phenotypes as risk factors, including CCR2 on CD14+ CD16+ monocytes, Transitional AC, CD19 on IgD− CD38−, and CD3 on HLA DR + CD4+; two protective, such as naive-mature B cell AC and NK AC.
- Malignant thymoma distinct associations: Seven risk phenotypes, e.g., Memory B cell %B cell, CD20− AC, CD3 on CD28+ CD45RA− CD8br, CD28 on CD28+ CD4+, CCR2 on CD14+ CD16+ monocyte, CD80 on plasmacytoid DC, and CD80 on CD62L+ plasmacytoid DC; three protective, including IgD+ CD24− %B cell, CD20 on IgD− CD38dim, and CD16 on CD14+ CD16+ monocyte, differentiating from benign profiles.
- Shared and differential mechanisms: CCR2 on CD14+ CD16+ monocytes causally promotes both benign and malignant thymomas, highlighting a potential progression biomarker amid distinct immune roles in tumor microenvironments.
- Immunotherapy Implications: Genetic evidence identifies avenues for targeted therapies, such as enhancing protective NK AC in benign cases or inhibiting risk factors like CD80 on DCs in malignant thymomas, advancing precision immunotherapy for thymic tumors.

## Materials and methods

### Study design

**Figure 1.**
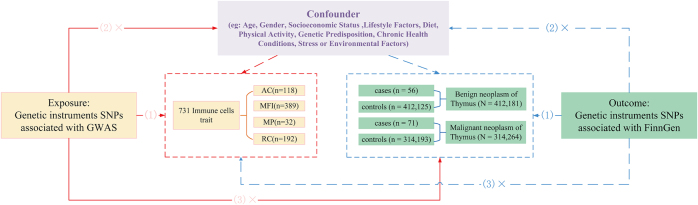
Design principles of MR study.

Our study followed the STROBE-MR statement used to report MR research^[25]^. We confirm that this study was reported in accordance with the reporting recommendations for tumor marker prognostic studies (REMARK) guidelines^[26]^. We utilized exposure and outcome data obtained from GWAS databases. The analyzed data were previously published and publicly available. The OpenGWAS database (https://gwas.mrcieu.ac.uk/) is a comprehensive platform that integrates genome-wide association study (GWAS) data on a wide range of diseases, genes, and phenotypic traits. Through a two-sample MR analysis, we explored the causal relationship between 731 immune cell traits and benign or malignant thymic tumors. A flowchart of the study design is shown in Figure 1. Outcome classifications accounted for WHO and ICD-O standards, including histologic heterogeneity, as detailed in the outcome data sources below. MR analysis requires fulfillment of the following three assumptions: (1). Relevance hypothesis: the selected instrumental variables (IVs) must be strongly associated with immune cell phenotypes (exposure factors); (2). Independence hypothesis: the chosen IVs should not be associated with the outcome variables (benign and malignant thymic tumors) or other confounding factors; (3). The IVs must influence benign and malignant thymic tumors through their effects on immune cell phenotypes^[27]^.

### Data sources for exposure data

GWAS data for 731 immunophenotypic profiles were extracted from the GWAS catalog spanning GCST90001391 to GCST90002121^[28]^. The GWAS involved a cohort of 3757 Europeans, with no overlap between the cohorts. A high-density array containing approximately 22 million single nucleotide polymorphism (SNPs) based on Sardinian reference sequence panels was used to examine correlations while controlling for covariates, such as age, age squared, and sex. The GWAS dataset included measurements of the median fluorescence intensity (MFI), indicating surface antigen levels (*n* = 389), absolute cell counts (AC; *n* = 118), morphological parameters (MP; *n* = 32), and relative cell counts (RC; *n* = 192). The immune cell types analyzed included the B cell; cDC; T cell; monocyte; bone marrow cell; T, B, and natural killer cells (TBNK); and Treg maturation stages.

### Data sources for outcome data

GWAS data on thymus gland malignant and benign tumors were obtained from https://storage.googleapis.com/finngen-public-data-r10/summary_stats/finngen_R10_C3_THYMUS_EXALLC.gz and https://storage.googleapis.com/finngen-public-data-r10/summary_stats/finngen_R10_CD2_benign_thymir.gz. The numbers in GWAS data points related to thymic malignancies were as follows: C3_THYMUS_EXALLC, sample size (*N* = 314 264), cases (*n* = 71), and controls (*n* = 314 193). The numbers in the GWAS data for benign thymic tumors were as follows: CD2_BENIGN_THYMUS, sample size (*N* = 412 181), cases (*n* = 56), and controls (*n* = 412 125). These classifications utilized ICD-O codes, with benign tumors (CD2_BENIGN_THYMUS) primarily encompassing code 0/1 variants (e.g., lipofibroadenoma, micronodular thymoma) and malignant (C3_THYMUS_EXALLC), including code 3 thymomas (types A–B3 with uncertain behavior) and heterogeneous carcinomas (e.g., squamous cell, sarcomatoid)^[3]^.

### Selection of instrumental variables (IVs)

To guarantee the robustness of the instrumental variables, we employed stringent thresholds (*P* < 5 × 10^−8^) to identify SNPs that were significantly associated with the presumed risk factors in published European GWAS^[28]^. We employed PLINK clustering to estimate the linkage imbalance between these SNPs for each risk factor using the 1000 genome European reference panel^[29]^. Finally, we employed MR analysis to incorporate SNPs with the largest impact on immune traits based on chain imbalance, employing standards, such as R^2^ > 0.001 and an aggregation window of <10 000 kb^[30]^. In the framework of the two-sample setup, we harmonized the summary statistics and removed palindromes and incompatible alleles, thereby strengthening the dependability of our instrumental variable selection. In addition, the proportion of phenotypic variation explained (PVE) and F-statistics were calculated for each IV to assess the strength of the IV and avoid weak instrumental bias. SNPs with an F-statistic value of <10 were determined to be weak instruments and were subsequently excluded from the IVs. F-statistic was estimated using the formula: F = R^2^ (N^2^)/(1 − R^2^), where R^2^ was the proportion of phenotypic variation explained by the SNP and N was the sample size of the GWAS of SNPs with the trait^[31]^. The R^2^ values were estimated using the formula: R^2^ = 2 × EAF × (1 − EAF) × β^2^, where EAF was the effect allele frequency of the SNP and β was the estimated effect of SNP on trait.

### MR analysis and sensitivity analysis

All analyses were performed using the R 3.4.3 software (http://www.Rproject.org). To evaluate the causal association between the 731 immunophenotypes and benign and malignant thymus tumors, inverse variance weighting (IVW), MR-Egger, weighted median, simple mode, and weighted mode approaches were performed using the MR package (version 0.4.3)^[32–35]^. Cochran’s Q statistic and corresponding *P-*values were used to test for heterogeneity among the selected IVs^[29]^. If the null hypothesis was rejected, random effects IVW was used instead of fixed effects IVW^[36]^. To exclude the effect of horizontal pleiotropy, a common method (i.e., MR-Egger) was used, which implies the presence of horizontal multiplicity if the intercept term is significant^[27]^. Furthermore, the powerful MR pleiotropy residual sum and outlier (MR-PRESSO) method was used to exclude possible horizontal pleiotropic outliers that could substantially affect the estimation results in the MR-PRESSO package^[37]^. Sensitivity analyses were conducted in the following sequence: (1) heterogeneity assessment using Cochran’s Q statistic (*P* < 0.05); (2) pleiotropy detection with MR-Egger intercept (*P* > 0.05) and MR-PRESSO global test (*P* > 0.05); (3) robustness evaluation via leave-one-out analysis and funnel plots to confirm symmetry and absence of influential outliers. Scatter and funnel plots were constructed. The scatter plots showed that the results were not affected by outliers. Funnel plots demonstrated the robustness of the correlations and the absence of heterogeneity.

## Results

### Exploring the causal role of immunophenotypes on benign neoplasm of thymus

**Figure 2.**
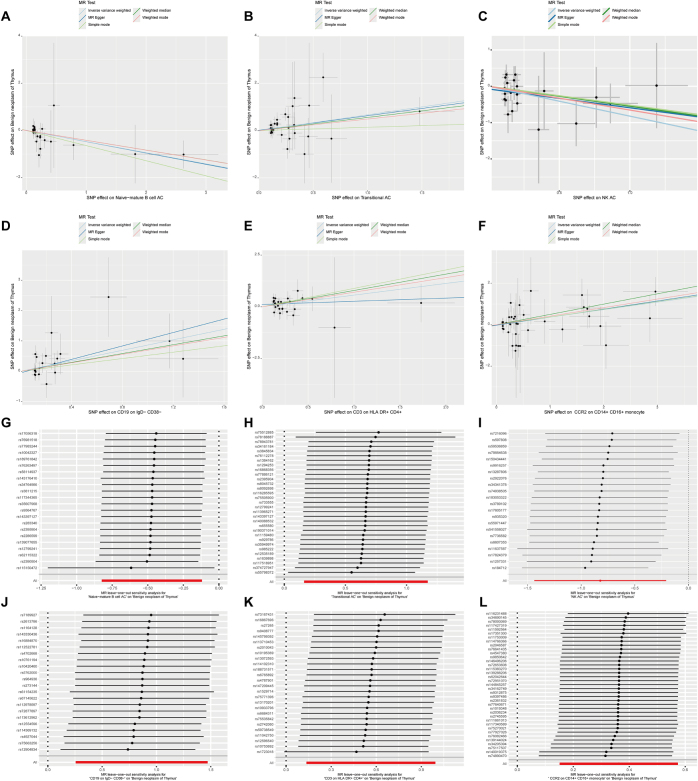
Scatter plots and leave-one-out test results for the causal relationship between six immune cell phenotypes and benign thymic tumors. The scatter plots reveal a negative correlation between Naive-mature B cell AC (A) and NK AC (C) with benign thymic tumors, whereas a positive correlation is observed for Transitional AC (B), CD19 on IgD− CD38− (D), CCR2 on CD14+ CD16+ Monocytes (E), and CD3 on HLA-DR + CD4+ (F). The leave-one-out causal relationship plots illustrate the association of Naive-mature B cell AC (G), transitional AC (H), NK AC (I), CD19 on IgD− CD38− (J), CCR2 on CD14+ CD16+ Monocytes (K), and CD3 on HLA-DR + CD4+ (L) with benign thymic tumors.

To explore the causal effects of immunophenotypes on benign thymic neoplasms, we performed a two-sample MR analysis and used the IVW method as the primary analysis. We identified significant associations between six immune phenotypes and benign thymic tumors (*P* < 0.001 for IVW), of which four were harmful to benign neoplasm of thymus: C-C motif chemokine receptor 2 (CCR2) on CD14+ CD16+ Monocyte (Monocyte panel), Transitional AC (B cell panel), CD19 on IgD− CD38− (B cell panel), and CD3 on HLA DR + CD4+ (T cell panel); and two were protective to benign neoplasm of thymus: naive-mature B cell AC (B cell panel) and NK AC (NK cell panel). Scatter plots were created to visualize the relationships between the exposure factors and outcome variables, illustrating the effect size of each SNP. A leave-one-out analysis was performed to assess the influence of each instrumental variable on the overall outcome (Fig. 2). Funnel plots indicated a symmetric distribution of the SNPs, supporting the robustness of the results (Supplementary Digital Content Figure 1, available at: http://links.lww.com/MS9/B32). Heterogeneity analysis was used to assess variation among the instrumental variables. Both MR-Egger regression and the IVW method produced *P* > 0.05 across all datasets, indicating no significant variability in causal effects. MR-PRESSO regression analysis detected directional horizontal pleiotropy. All *P*-values exceeded 0.05, indicating no horizontal pleiotropy, further supporting the accuracy of the causal inference (Supplemental Digital Content Table 1, available at: http://links.lww.com/MS9/B28).

Specifically, by using the IVW method, the odds ratio (OR) of naive-mature B cell AC on benign neoplasm of thymus risk was estimated to be 0.624 (95% CI: 0.438–0.887, *P* = 0.0087), the OR of NK AC on benign neoplasm of thymus risk was estimated to be 0.439 (95% CI: 0.237–0.812, *P* = 0.0087), the OR of CCR2 on CD14+ CD16+ Monocyte on benign neoplasm of thymus risk was estimated to be 1.437 (95% CI: 1.163–1.775, *P* = 0.0007), the OR of transitional AC on benign neoplasm of thymus risk was estimated to be 1.950 (95% CI: 1.176–3.232, *P* = 0.0095), the OR of CD19 on IgD− CD38− on benign neoplasm of thymus risk was estimated to be 2.372 (95% CI: 1.292–4.355, *P* = 0.0053), and the OR of CD3 on HLA DR + CD4+ on benign neoplasm of thymus risk was estimated to be 1.759 (95% CI: 1.145–2.702, *P* = 0.0099; Fig. 4; Supplemental Digital Content Table 2, available at: http://links.lww.com/MS9/B29).

### Exploring the causal role of immunophenotypes on malignant neoplasm of thymus

**Figure 3.**
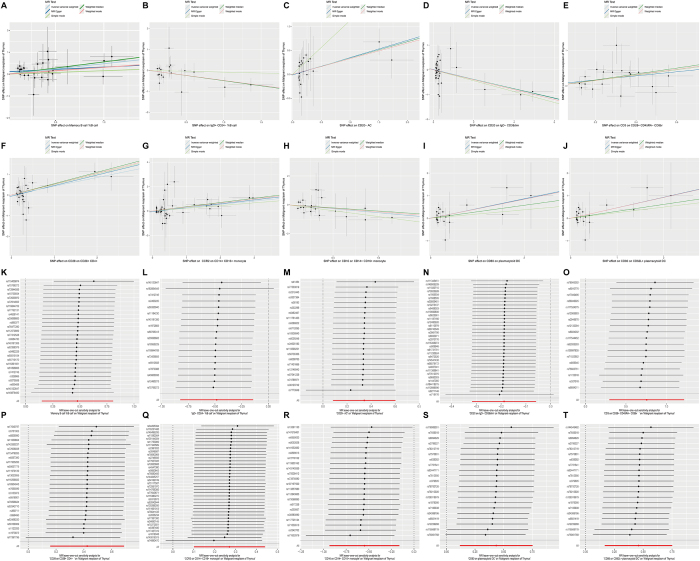
Scatter plots and leave-one-out test results for the causal relationship between ten immune cell phenotypes and malignant thymic tumors. The scatter plots show a negative correlation between IgD+ CD24− %B cells (B), CD20 on IgD− CD38dim (D), and CD16 on CD14+ CD16+ monocytes (H) with malignant thymic tumors, whereas a positive correlation is observed for Memory B cell %B cells (A), CD20− AC (C), CD3 on CD28+ CD45RA− CD8bright (E), CD28 on CD28+ CD4+ (F), CCR2 on CD14+ CD16+ monocytes (G), CD80 on plasmacytoid DC (I), and CD80 on CD62L+ plasmacytoid DC (J). The leave-one-out causal relationship plots show the association of Memory B cell %B cells (K), IgD+ CD24− %B cells (L), CD20− AC (M), CD20 on IgD− CD38dim (N), CD3 on CD28+ CD45RA− CD8bright (O), CD28 on CD28+ CD4+ (P), CCR2 on CD14+ CD16+ monocytes (Q), CD16 on CD14+ CD16+ monocytes (R), CD80 on plasmacytoid DC (S), and CD80 on CD62L + plasmacytoid DC (T) with malignant thymic tumors.

To explore the causal effects of immunophenotypes on malignant thymic neoplasms, we performed a two-sample MR analysis and used the IVW method as the primary analysis. We identified significant associations between ten immune phenotypes and malignant thymic tumors (*P* < 0.001 for IVW), of which, seven were harmful to malignant neoplasm of thymus: Memory B cell %B cell (B cell panel), CD20− AC (B cell panel), CD3 on CD28+ CD45RA− CD8br (T cell panel), CD28 on CD28+ CD4+ (T cell panel), CCR2 on CD14+ CD16+ monocyte (monocyte panel), CD80 on plasmacytoid DC (DC panel), and CD80 on CD62L + plasmacytoid DC (DC panel); and three were protective to malignant neoplasm of thymus:. IgD+ CD24− %B cell (B cell panel), CD20 on IgD− CD38dim (B cell panel), and CD16 on CD14+ CD16+ monocyte (monocyte panel). Scatter plots were created to visualize the relationships between the exposure factors and outcome variables, illustrating the effect size of each SNP. A leave-one-out analysis was performed to assess the influence of each instrumental variable on the overall outcome (Fig. 3). Funnel plots indicated a symmetric distribution of the SNPs, supporting the robustness of the results (Supplementary Digital Content Figure 2, available at: http://links.lww.com/MS9/B32). Heterogeneity analysis was used to assess variation among the instrumental variables. Both MR-Egger regression and the IVW method produced *P* > 0.05 across all datasets, indicating no significant variability in causal effects. MR-PRESSO regression analysis detected directional horizontal pleiotropy. All *P*-values exceeded 0.05, indicating no horizontal pleiotropy, further supporting the accuracy of the causal inference (Supplemental Digital Content Table 3, available at: http://links.lww.com/MS9/B30).

**Figure 4.**
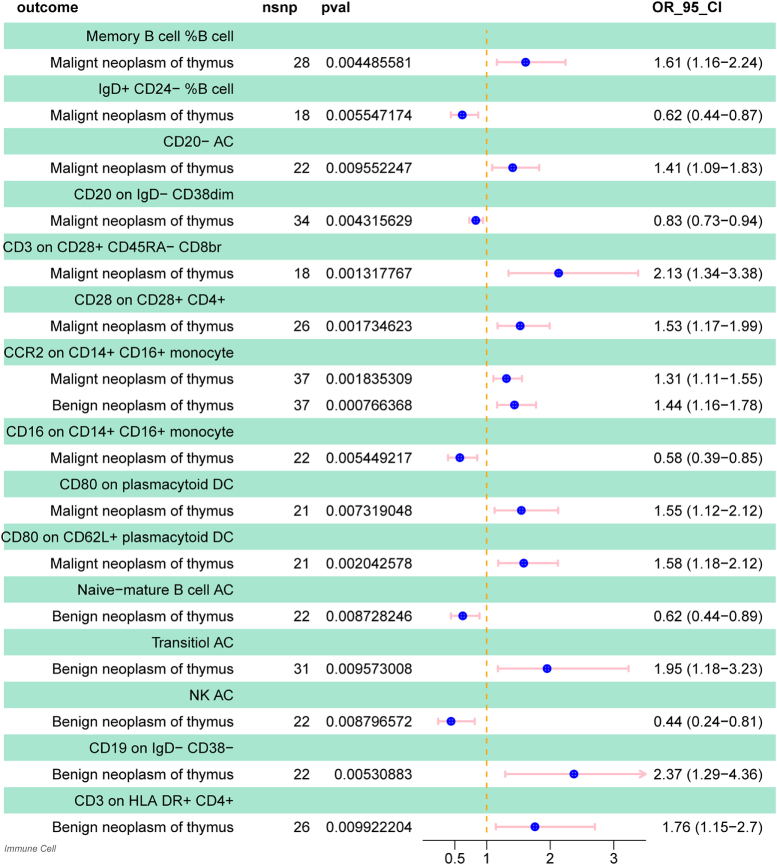
Forest plot showing the causal relationship between specific immune cell phenotypes and benign and malignant thymic tumors (IVW method). Phenotypes are stratified by outcome (benign neoplasm of the thymus; malignant neoplasm of the thymus). Data columns include number of SNPs as instrumental variables, *P*-value for the IVW estimate, and odds ratio (OR) with 95% confidence interval (CI). Point estimates are represented as squares (size proportional to inverse variance weight), with horizontal lines denoting 95% CIs. OR > 1.0 (point to the right of the reference line) indicates increased risk; OR < 1.0 (to the left) indicates protective effect. Statistical significance is defined as *P* < 0.05.

Specifically, by using the IVW method, the ORs of Memory B cell %B cell, IgD+ CD24− %B cell, CD20− AC, CD20 on IgD− CD38dim, CD3 on CD28+ CD45RA− CD8br, CD28 on CD28+ CD4+, CCR2 on CD14+ CD16+ monocyte, CD16 on CD14+ CD16+ monocyte, CD80 on plasmacytoid DC, and CD80 on CD62L + plasmacytoid DC on malignant neoplasm of thymus risk were estimated to be 1.612 (95% CI: 1.160–2.242, *P* = 0.0045), 0.617 (95% CI: 0.438–0.868, *P* = 0.0055), 1.409 (95% CI: 1.087–1.826, *P* = 0.0096), 0.829 (95% CI: 0.728–0.943, *P* = 0.0043), 2.131 (95% CI: 1.343–3.382, *P* = 0.0013), 1.527 (95% CI: 1.172–1.991, *P* = 0.0017), 1.311 (95% CI: 1.106–1.554, *P* = 0.0018), 0.578 (95% CI: 0.393–0.851, *P* = 0.0054), 1.545 (95% CI: 1.124–2.124, *P* = 0.0073), and 1.583 (95% CI: 1.182–2.120, *P* = 0.0020), respectively (Fig. 4; Supplementary Digital Content Table. 4, available at: http://links.lww.com/MS9/B31).

## Discussion

Based on extensive publicly available genetic data, we identified causal relationships between 731 immune cell traits and both benign and malignant thymic tumors, and explored the crucial roles of specific immune phenotypes in their development and progression. This study examined four categories of immune traits (MFI, RC, AC, and MP) and revealed six immune phenotypes with significant causal effects on benign thymic tumors and ten phenotypes affecting malignant thymic tumors. These findings emphasize the distinct functional roles of immune cells in benign and malignant thymic tumors, and provide novel insights into the immunological mechanisms that drive thymic tumor development. These distinctions align with the WHO and ICD-O classifications of thymomas, including histologic heterogeneity, as detailed in the Introduction, underscoring potential implications for differential diagnosis and treatment strategies^[2]^.

Our study found that naïve-mature B cell and NK ACs were associated with a reduced risk of benign thymomas. Immature B cells produced in the bone marrow are mostly self-reactive and capable of binding to self-antigens. Conversely, mature B cells, undergo peripheral organ maturation and can elicit functional immune responses against antigens^[38,39]^. Both antigen-mediated and functionally enhanced B cells can be influenced by signals from receptor-binding cytokines, chemokines, or other factors and participate in tumor cell clearance through antibody-dependent cellular cytotoxicity (ADCC) and complement activation^[40]^. NK cells are a crucial component of the innate immune system and play a key role in maintaining immune homeostasis by non-specifically killing target cells, such as virus-infected and tumor cells^[41]^. Studies have shown that NK cell-mediated immunotherapy is a safe and effective treatment for patients with advanced leukemia. One of the major mechanisms limiting NK cell activity is the immunosuppressive TME. NK cell metabolism is impaired in the TME, potentially due to inhibitory cytokines (e.g., TGF-β) and tumor-derived metabolic end-products, such as lactate, which suppress NK cell function^[42]^. Additionally, tumor-associated macrophages and myeloid-derived suppressor cells can impair NK cell cytotoxicity by interfering with NK AC signaling^[43]^. The study also revealed that transitional AC, CD19 on IgD^−^ CD38^−^, and CD3 on HLA-DR^+^ CD4^+^ might be risk factors for benign thymomas. Transitional B cells are an intermediate stage of B cell migration from the bone marrow to the periphery and are essential for the maturation process. In the TME, transitional B cells are activated by tumor-associated antigens and differentiate into antibody-secreting plasma or memory B cells^[44,45]^. These cells regulate immune responses by secreting anti-inflammatory factors, suppressing antitumor T cell activity, and forming immune barriers with tumor-associated lymphoid tissues, aiding tumor immune evasion^[46]^. CD19 is a core signaling molecule in B cells that functions with the B cell receptor (BCR) to enhance responses to antigen stimulation^[47]^. CD19 on IgD^−^ CD38^−^ typically represents functional memory B cells, which have shown notable associations in studies on breast and gastrointestinal cancers^[48,49]^. During tumor development, these cells encounter specific antigens in lymphoid tissues, and with the help of T cells, differentiate and produce antibodies that counteract tumor proliferation and metastasis. CD3 is a core component of the T cell receptor complex, indicating the presence of T cells. CD3 on HLA-DR^+^ CD4^+^ cells generally indicate activated helper T cells, which, upon antigen stimulation, secrete cytokines, such as IFN-γ and IL-2, playing a crucial role in regulating and enhancing immune responses^[50,51]^. Research on CD19 on IgD^−^ CD38^−^ and CD3 on HLA-DR^+^ CD4^+^ as prognostic and predictive biomarkers is ongoing. Therefore, exploring the changes in immune cell composition during the disease process could provide new directions for future clinical immunotherapy strategies for benign thymomas. This highlights the unique immune environment of thymomas, characterized by thymic epithelial disruption and autoimmunity links, differing from PD-L1-dominant evasion in lung cancer or B cell dysregulation in lymphoma^[21–23]^.

In the study of malignant thymomas, 10 specific immune cell phenotypes were identified, among which 3 (IgD^+^ CD24^−^ %B cells, CD20 on IgD^−^ CD38dim, and CD16 on CD14^+^ CD16^+^ monocytes) exhibited protective effects, whereas 7 [Memory B cells %B cells, CD20^−^ AC, CD3 on CD28^+^ CD45RA^−^ CD8br, CD28 on CD28^+^ CD4^+^ T cells, CCR2 on CD14^+^ CD16^+^ monocytes, CD80 on plasmacytoid dendritic cells (pDCs), and CD80 on CD62L^+^ plasmacytoid dendritic cells] demonstrated significant pro-tumorigenic roles. These findings provide crucial insights into immunological differences between benign and malignant thymomas. IgD^+^ CD24^−^ B cells may function as low-activation antigen recognition cells, transmitting signals via the BCR to trigger downstream signaling cascades, such as the PI3K/Akt and NF-κB pathways^[52]^. CD20, a B cell-specific surface molecule, is closely associated with B cell activation and survival. B cells lacking CD20 expression in early developmental stages, are typically differentiated into plasma cells, or are associated with pathological states, such as malignant transformation^[53]^. CD14, as a receptor for lipopolysaccharide (LPS), participates in pathogen recognition and signal transduction, whereas CD16 (also known as Fcγ receptor III) mediates antibody-dependent phagocytosis. CD14^+^ CD16^+^ monocytes primarily regulate inflammation by producing IL-10 and TGF-β, aiding in tissue repair post-inflammation^[54,55]^.

The role of memory B cells (MBCs) in disease mechanisms is currently the focus of immunotherapy research. These long-lived cells differentiate from activated B cells after antigen exposure, and upon re-encountering antigens, they rapidly become antibody-secreting cells or re-enter germinal centers to further diversify and enhance antigen affinity^[56]^. The CD3 on CD28^+^ CD45RA^−^ CD8br has been shown to have a significant causal relationship with esophageal cancer, although its mechanism in thymoma remains largely unexplored^[57]^. Studies have reported that CD4+ T cells express Notch ligands on their surface upon early activation by soluble anti-CD3 and CD28 antibodies, with ligands, such as DLL1, DLL4, and JAG1^[58]^. Plasmacytoid dendritic cells (pDCs) are a specialized dendritic cell subtype capable of efficiently secreting type I interferons (e.g., IFN-α), playing a crucial role in regulating the immune microenvironment^[59]^. In the context of autoimmune tolerance, pDCs may reduce self-reactive T-cell activity via the CD80–CTLA-4 pathway. CD62L (L-selectin), an adhesion molecule, facilitates the migration of immune cells from the bloodstream to peripheral lymphoid organs, such as the lymph nodes, initiating primary immune responses^[60]^. Although clinical studies on CD80 expression on CD62L^+^ pDCs are scarce, the findings of the study on its pro-tumorigenic effects in malignant thymomas provide a theoretical foundation for future targeted immunotherapy.

Our study revealed that the expression of CCR2 on CD14^+^ CD16^+^ monocytes promotes both benign and malignant thymomas. CCR2, a critical member of the chemokine receptor family, is primarily expressed on the surface of monocytes, macrophages, and some T cells and plays a pivotal role in shaping and regulating the tumor immune microenvironment. Previous research has demonstrated that CCR2, by binding to its ligand CCL2, drives the migration of CD14+ CD16+ monocytes from the peripheral blood to tumor sites, constituting a key pathway for immune cell recruitment to the TME^[61]^. Tumor cells, fibroblasts, and stromal cells often secrete high levels of CCL2, which attracts CCR2 + monocytes to the TME. This mechanism provides the TME with a notable supply of pro-inflammatory or immunosuppressive cells, which are essential for tumor growth in both benign and malignant thymomas^[62,63]^. Thus, CCR2 on CD14^+^ CD16^+^ monocytes emerges as a critical driver of the transition from benign to malignant thymomas. These findings suggest that benign and malignant thymomas may be driven by distinct immunological mechanisms, highlighting CCR2 as a potential biomarker for molecular subtyping and a foundational target for the development of stratified therapeutic strategies. Furthermore, these immune cell dynamics may be further influenced by the tumor extracellular matrix, as explored below.

The tumor extracellular matrix plays a pivotal role in modulating immune cell behavior within the TME, influencing invasion, polarization, and proliferation of key phenotypes identified in our study^[64,65]^. Composed of collagens, glycoproteins, and proteoglycans, the oncomatrix can alter matrix stiffness and composition, promoting chemokine signaling such as CCR2-CCL2 interactions that drive monocyte recruitment and polarization toward pro-tumorigenic states, as evidenced by our MR findings (OR: 1.437 for benign and 1.311 for malignant thymomas, *P* < 0.002). For instance, in thymic tumors, oncomatrix remodeling may enhance the infiltration of CD14^+^ CD16^+^ monocytes, exacerbating immune evasion and tumor progression while impairing protective cells like NK cells through metabolic suppression. This matrix-immune interplay underscores the distinct roles of immune phenotypes in benign versus malignant thymomas, providing mechanistic context to our genetic associations and highlighting potential targets for matrix-modulating therapies alongside immunotherapy.

To date, studies investigating the role of immune cells in thymic tumors are limited. This study introduced a notable innovation by employing MR analysis, a method that uses genetic variants as instrumental variables to overcome confounding factors and reverse the causality challenges inherent in traditional observational studies. This approach enables reliable causal inferences between immune cell phenotypes and thymic tumors. Unlike previous research, which primarily focused on descriptive analyses exploring specific immune cells or signaling pathways within the TME, this study adopted a comprehensive and systematic approach to reveal the diverse functional patterns of immune cells in benign and malignant thymomas. However, this study had some limitations. First, the two-sample MR analysis primarily utilized the inverse variance-weighted (IVW) method, whereas the results from alternative MR methods showed certain inconsistencies. Even after conducting rigorous sensitivity analyses, potential pleiotropy between immune cells and thymomas cannot be entirely excluded^[27]^. Second, the study relied on data from European cohorts, which limits the generalizability of the findings to other ethnic groups^[20]^. Future research incorporating large-scale GWAS data from Asian, African, and other populations could provide a more inclusive perspective to address this limitation. Third, the low number of thymoma cases (benign: 56; malignant: 71) represents a critical limitation in MR power, potentially increasing the risk of false positives or negatives, although mitigated by strong instrumental variables (F-statistics > 10)^[31]^. Finally, although this study identified causal relationships between immune cell phenotypes and thymic tumors, it did not explore the underlying biological mechanisms that drive these associations (e.g., the role of transitional B cells or CD80+ DCs), which future studies should prioritize to confirm these associations.

Our findings provide genetic evidence that can inspire advancements in tumor immunotherapy, a rapidly evolving field with recent breakthroughs in checkpoint inhibitors and cell therapies^[66]^. For instance, the protective effects of NK AC in benign thymomas suggest potential for NK cell-enhancing therapies, while risk factors like CD80 on plasmacytoid DCs in malignant cases highlight targets for inhibition, such as CD80–CTLA-4 pathway modulators. Combining these insights with overall progress in oncology immunotherapy could guide screening of therapeutic targets, optimize patient stratification based on immune phenotypes, and predict responses in thymoma’s highly immune-relevant TME. Future research should validate these in clinical trials, explore dynamic immune changes over time, and integrate multi-omics data to advance precision immunotherapy for thymic tumors.

In summary, this study used robust MR analysis to establish the causal roles of immune cells in both benign and malignant thymomas. These findings deepen our understanding of the immunological mechanisms underlying thymic tumors and provide a theoretical foundation for their precise diagnosis, treatment, and prognostic assessment. Future studies should investigate dynamic changes in immune phenotypes, validate findings across diverse populations, and develop targeted therapies based on newly identified immune cell phenotypes. These efforts will further advance thymic tumor immunology and precision medicine.

## Conclusion

In summary, a comprehensive MR analysis revealed causal associations between various immune cell phenotypes and benign and malignant thymomas, emphasizing the intricate interactions between the immune system and thymic tumors. Notably, four immune cell phenotypes were associated with a significantly increased risk of benign thymomas, whereas two were associated with a decreased risk. For malignant thymomas, seven immune cell phenotypes were associated with a higher risk, whereas three were associated with a lower risk. This genetic perspective provides novel insights into the immunological mechanisms of benign and malignant thymomas and may guide the development of new immunotherapeutic approaches.

## Data Availability

Our analysis used publicly available GWAS (https://gwas.mrcieu.ac.uk/) summary statistics. Genetic association estimates for benign and malignant thymomas were obtained from the FinnGen consortium (www.finngen.fi/en). The original contributions of this study are included in the article/supplementary material section. Further inquiries can be directed to the corresponding author.
